# Fibroblast growth factor receptors in cancer: genetic alterations, diagnostics, therapeutic targets and mechanisms of resistance

**DOI:** 10.1038/s41416-020-01157-0

**Published:** 2020-12-03

**Authors:** Melanie A. Krook, Julie W. Reeser, Gabrielle Ernst, Hannah Barker, Max Wilberding, Gary Li, Hui-Zi Chen, Sameek Roychowdhury

**Affiliations:** 1 Center for Clinical and Translational Science, The Ohio State University, Columbus, OH USA; 2 Department of Internal Medicine, The Ohio State University Comprehensive Cancer Center, Columbus, OH USA; 3 QED Therapeutics Inc., San Francisco, CA USA

**Keywords:** Cancer, Cancer

## Abstract

Fibroblast growth factor receptors (FGFRs) are aberrantly activated through single-nucleotide variants, gene fusions and copy number amplifications in 5–10% of all human cancers, although this frequency increases to 10–30% in urothelial carcinoma and intrahepatic cholangiocarcinoma. We begin this review by highlighting the diversity of *FGFR* genomic alterations identified in human cancers and the current challenges associated with the development of clinical-grade molecular diagnostic tests to accurately detect these alterations in the tissue and blood of patients. The past decade has seen significant advancements in the development of FGFR-targeted therapies, which include selective, non-selective and covalent small-molecule inhibitors, as well as monoclonal antibodies against the receptors. We describe the expanding landscape of anti-FGFR therapies that are being assessed in early phase and randomised controlled clinical trials, such as erdafitinib and pemigatinib, which are approved by the Food and Drug Administration for the treatment of *FGFR3*-mutated urothelial carcinoma and *FGFR2*-fusion cholangiocarcinoma, respectively. However, despite initial sensitivity to FGFR inhibition, acquired drug resistance leading to cancer progression develops in most patients. This phenomenon underscores the need to clearly delineate tumour-intrinsic and tumour-extrinsic mechanisms of resistance to facilitate the development of second-generation FGFR inhibitors and novel treatment strategies beyond progression on targeted therapy.

## Background

**Fig. 1:**
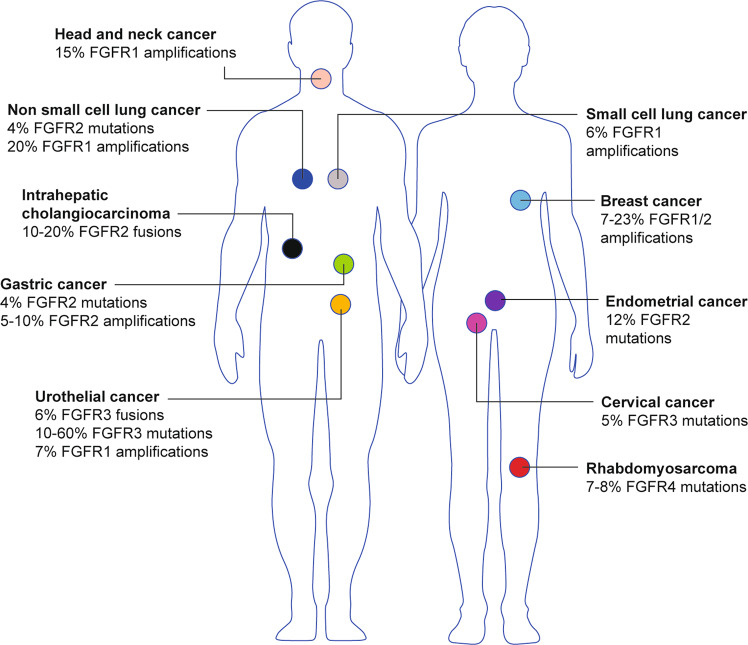
Cancer types that harbour alterations in *FGFR*. *FGFR* alterations, including single-nucleotide variants (SNVs), fusions and copy number amplifications (CNAs) have been frequently detected in multiple types of human cancer at varying percentages.

The fibroblast growth factor receptor (FGFR) family of receptor tyrosine kinases consists of four transmembrane receptors, FGFR1–4.^1^ Each receptor contains three extracellular immunoglobulin (Ig)-like binding domains, followed by a transmembrane domain and an intracellular domain constituting a two-part tyrosine kinase.^1^ Twenty-two known fibroblast growth factor (FGF) ligands exist, yet only 18 of these ligands^2^ interact with, and induce the dimerisation of, these four receptors to stimulate their kinase activity and activate downstream signalling pathways through the intracellular domain. These pathways include the extracellular signal-regulated kinase (ERK)/mitogen-activated protein kinase (MAPK) pathway, which promotes cell survival, proliferation, development, angiogenesis and differentiation.^3–6^ Consequently, aberrations in *FGFR1–4*—including single-nucleotide variants (SNVs), gene rearrangements or fusions, and copy number amplifications (CNAs)—are detected in 5–10% of all human cancers, although some types, such as urothelial cancer and intrahepatic cholangiocarcinoma (iCCA), display an increased (10–30%) frequency of *FGFR* aberrations (Fig. 1).^3,7–9^ Given the diversity of *FGFR* alterations, in particular with fusions as drivers, in solid tumour and haematological cancers, there is an emerging need for clinical-grade molecular diagnostic tests to accurately detect these aberrations in both tumour tissues and blood samples. The repertoire of FGFR-targeted therapies has expanded to include non-selective tyrosine kinase inhibitors (TKIs), selective TKIs, covalent TKIs, monoclonal antibodies and antibody–drug conjugates (ADCs), and FGF ligand traps. Two anti-FGFR therapies have recently been approved by the Food and Drug Administration (FDA), including erdafitinib for *FGFR3*-altered urothelial cancer and pemigatinib for *FGFR2*-fusion cholangiocarcinoma.^10,11^ While the majority of patients are initially sensitive to FGFR-targeted therapies, many develop acquired resistance ultimately resulting in disease progression and discontinuation of therapy. Secondary genomic alterations responsible for TKI resistance are becoming more defined, such as gatekeeper mutations in the *FGFR* kinase domain. Additional mechanistic studies are clearly needed to fully elucidate the complexities of acquired resistance, including undefined molecular contributions from the tumour stroma and immune system. Only a full understanding of how different cell types and their interplay behave in various cellular contexts that mediate resistance will lead to the development of second and subsequent generation of TKIs and combinatorial therapies. In this review, we will catalogue the diversity of *FGFR* alterations in human cancers, discuss the challenges associated with molecular diagnostic tests being developed to detect these alterations for clinical actionability, review the diverse portfolio of first-generation anti-FGFR therapies and summarise current known mechanisms of acquired resistance.

## *FGFR* alterations in cancer

Advances in sequencing technologies in recent years have led to the discovery of diverse *FGFR* genomic alterations that have been shown to occur at varying frequencies across numerous tumour types. Helsten et al.^3^ reported that of the 7.1% of *FGFR1–4*-altered cancers in their cohort of 4853 tumours, 66% of the aberrations were due to CNAs, while 26% were SNVs, and 8% were gene rearrangements or fusions. In a separate retrospective analysis of 274,694 patient tumour samples, genomic alterations in *FGFR1–3* (FGFR4 was not assessed) were detected by next-generation sequencing in 2.3% of patient specimens, of which 64.8% were SNVs and 35.9% were rearrangements.^12^ In both studies, almost all tumour types were found to have *FGFR* alterations, but those with the highest alteration frequency included urothelial cancer, cholangiocarcinoma, endometrial cancer, squamous lung cancers, breast cancer and cervical cancer.^3,12^ In this section, we catalogue the diversity of FGFR SNVs, gene fusions and CNAs.

### Single-nucleotide variants

Somatic activating *FGFR1–4* SNVs can cause the receptor to be constitutively active by conferring increased dimerisation, increased kinase activity or enhanced affinity for FGF ligands.^1^ Unlike other kinases, such as epidermal growth factor receptor (EGFR) and vascular endothelial growth factor receptor (VEGFR), in which activating SNVs tend to occur exclusively within the kinase domain, SNVs in *FGFR1–4* have been reported in the extracellular domain, the transmembrane domain and the intracellular kinase domain.^12,13^ A recent large retrospective analysis of next-generation sequencing (NGS) data from >250,000 patient samples identified over 250 unique SNVs distributed throughout the different domains of FGFR1–3, highlighting the vast diversity of FGFR SNVs seen across cancer types.^12^

*FGFR1* SNVs are rare and have been reported in fewer cases of cancer in comparison with *FGFR2* and *FGFR3*. The two most common activating mutations in *FGFR1* are N546K and K656E, both of which reside in the kinase domain and result in increased kinase activation and transformation in vitro.^3,14–16^ Although the functional consequence is unknown, the S125L mutation has been reported in both breast and gallbladder cancer.^17,18^

The majority of SNVs in *FGFR* have been reported to occur in *FGFR2* and are found at high frequencies in endometrial cancer (12%), non-small cell lung cancer (4%) and gastric cancer (4%).^1,19,20^ Interestingly, these somatic-activating *FGFR2* mutations predominantly occur in the transmembrane (Y375C, C382Y/R) and extracellular domains (S252W, W290C, P253R) rather than the kinase domain (N549H/K, K659E).^3^ Extensive in vitro and in vivo analyses of these mutations have revealed that their oncogenic potential is due to increased receptor–ligand binding affinity and receptor dimerisation. Furthermore, studies have revealed that these mutations are highly sensitive to FGFR inhibition.

Activating *FGFR3* mutations have been identified in 10–60% of urothelial carcinomas, predominately in low-grade tumours.^21^ The most frequent *FGFR3* SNVs were R248C and S249C occurring in the extracellular domain, as well as G370C and Y373C occurring in the transmembrane domain.^1^ The resulting cysteine residues from these mutations lead to ligand-independent dimerisation of the receptor.^22^ In addition, *FGFR3* mutations have been reported to occur in 5% of cervical carcinomas.^23^

*FGFR4* SNVs are notable for their prevalence in rhabdomyosarcoma, occurring in 7–8% of cases.^24^ Specifically, V550E, a gatekeeper mutation, and N535K both contribute to autophosphorylation and constitutive activation of the kinase.^25^ In a transcriptome screen of cancer cell lines, an *FGFR4* Y367C mutation was identified in the human breast cancer cell line, MDA-MB453.^26^ In vitro characterisation of this mutation showed that it promotes spontaneous dimerisation, resulting in constitutive receptor activation in a ligand-independent manner.^27^

In summary, broad analyses aimed at determining the frequency and type of somatic SNVs in *FGFR1–4* in human cancer have revealed a diverse spectrum of variants spanning the entire protein sequence. While the tumour-promoting activity of a subset of these variants have been demonstrated and validated in cell lines, the tumorigenic potential of other variants remains to be fully characterised. Furthermore, complementary pre-clinical studies designed to establish the sensitivity of *FGFR1–4* SNVs to various FGFR inhibitors may eventually guide the molecular stratification of patients and clinical selection of matched therapies.

### Gene fusions

*FGFR* gene fusions can occur through chromosomal rearrangements or translocations, leading to increased receptor dimerisation and activation, as well as the dysregulated expression of *FGFR* or its fusion partner gene.^1^ Fusions of *FGFR1–3* involving many different partner genes have been detected in a variety of cancers, including breast cancer, urothelial carcinoma, glioblastoma, head and neck squamous cell carcinoma, iCCA, low-grade glioma, lung adenocarcinoma, lung squamous cell carcinoma, ovarian cancer, prostate adenocarcinoma and thyroid carcinoma.^3^ Among these cancers, a majority of the *FGFR* fusions occur in-frame to produce a functional chimeric protein.^1^ Depending on whether the FGFR N terminus or C terminus is involved in the rearrangement, FGFR fusions have been classified as type I or type II, respectively. Type I fusions appear characteristic of rearrangements that occur in haematological malignancies, while type II fusions are more frequently detected in solid malignancies. In type I fusions, loss of the transmembrane or extracellular domain of FGFR leads to the incorrect localisation of these fusion proteins and dysregulated function. Instead of FGFR localising to the plasma membrane, depending on the fusion partner and the domains that are maintained, the fusion may localise to a different area of the cell with an altered level of kinase activity. The fusion CEP110-FGFR1 has been found to localise to the cytoplasm, which counteracts the expectation that it would lead to the centrosome based on the region of CEP110 retained in the fusion.^18^ This is in contrast with the type II fusions, in which the loss of the phospholipase-C-binding tyrosine at the C terminus leads to upregulated signal transduction.^28^ Ligand-independent receptor dimerisation or increased kinase activity in the fusion protein leads to the activation of downstream oncogenic pathways and malignant transformation.

Unfortunately, there is sparse information as to why *FGFR1–3* prefers to partner with specific genes or why certain gene partners appear more common than others (e.g. BicC family RNA-binding protein 1, *BICC1*). In addition, why gene fusions even occur is not fully understood. Multiple mechanisms have been proposed, but overall there is insufficient evidence to confirm that these fusion events occur as anything other than as a result of chance.^29^ In spite of this, several gene fusions have long been regarded as driver alterations, including *BCR–ABL* and rearrangements involving *ALK*, *ROS1* and *NTRK*. The notion of fusions as oncogenic drivers is further bolstered by the dramatic and often durable responses observed in patients with advanced solid and haematological malignancies treated with fusion-specific inhibitors, such as imatinib (*BCR–ABL*), brigatinib and alectinib (*ALK* and *ROS1*), as well as entrectinib and larotrectinib (*NTRK*).^30–34^ Wu et al.^35^ examined possible mechanisms for the oncogenic properties of *FGFR* fusions through in vitro studies of the known fusion partners *BICC1*, *TACC3* (transforming acidic coiled-coil containing protein 3), *CCDC6*, *BAIAP2L1*, *KIAA1976*, *CASP7*, *CIT* and *OFD1*, concluding that these partners bring about receptor oligomerisation and activate one of the *FGFR* kinase domains. In another study, Singh et al.^36^ reported that the fusion of FGFR3 with the *TACC3* in glioblastoma conferred constitutive phosphorylation and kinase activity, leading to mislocalisation of the mitotic spindles and aneuploidy, resulting in oncogenic transformation.

In the study of *FGFR*-altered cancers by Helsten et al.,^3^ fusions involving *FGFR2*/*FGFR3* and *TACC3* were the most commonly detected fusion event, followed by fusions involving *NPM1*, *TACC2* and *BICC1.* The *FGFR2* fusion partners *AFF3*, *CASP7* and *CCDC6* have been shown to aberrantly activate *FGFR2* in triple-negative breast cancer, while *FGFR3–TACC3* and *FGFR2–CIT* have been detected in lung cancer; the *FGFR3–TACC3* fusion also occurs in ~2% of patients with urothelial carcinoma, cervical squamous cell carcinoma and glioblastoma.^37^ Type I FGFR fusions, including *CNTRL–FGFR1*, *ZMYM2–FGFR2*, *BRC–FGFR1* and *ETV6–FGFR3*, have been detected in patients with acute myeloid leukaemia, acute lymphoid leukaemia and peripheral T cell lymphoma.^37^ In summary, FGFR fusions occur frequently in a variety of human cancers and confer oncogenic properties to the cells that harbour these fusions.

### Copy number amplifications

The most common genomic alteration in the FGFR family is gene amplification, with *FGFR1* and *FGFR4* having the highest frequencies of amplification seen in a study done by Helsten et al.^3^ *FGFR1* amplifications are common in multiple cancer types, including hormone-receptor positive (HR^+^), human epidermal growth factor receptor 2-positive (HER2^+^), and triple-negative breast cancer patients at frequencies of 23%, 27%, and 7%, respectively. *FGFR1* amplification in breast cancer has been associated with poor prognosis and disease relapse.^7^ In addition to breast cancer, *FGFR1* amplification has been detected in non-small cell lung carcinoma at 17%, small cell lung carcinoma at 6% and urothelial cancer at 7%.^1,3^ In the study done by Helsten et al.,^3^ of 343 patients with an *FGFR* alteration, 89% of *FGFR1* and 78% of *FGFR4* alterations were amplifications compared with *FGFR2* and *FGFR3* with frequencies of 49% and 30%, respectively.^3^ Although less common than *FGFR1* and *FGFR4*, *FGFR2* amplifications have also been detected in gastric and breast cancer.^1^ Interestingly, *FGFR2* amplifications have been associated with a C-terminal truncation of the gene, leading to an enhanced potential for oncogenic functions due to receptor malfunction of the internalisation mechanism. When comparing *FGFR1* with *FGFR2* amplification, it is important to note the differences in their amplicon structures: *FGFR2* is contained within a relatively short amplicon located around 10q26, whereas *FGFR1* amplification occurs within a large and longer amplicon containing multiple co-amplified genes within 8p11-12.^1,38^

Finally, despite CNA being the most common class of genomic alteration of *FGFR1–4*, CNA alone, in particular involving *FGFR1*, has proven inadequate as a predictive biomarker. Concern has been raised regarding the usefulness of gene amplification without correlating to mRNA or protein expression as selection for FGFR-targeted therapies.^39^ Generally, oncogene amplification is presumed to result in the upregulation of protein expression, which leads to ectopic protein function and ‘oncogene addiction’. However, a clinical study that selected lung cancer patients of all histologies for treatment with the multikinase inhibitor ponatinib based on *FGFR1* amplification and mRNA expression revealed a low rate of concordance between *FGFR1* amplification and actual mRNA (thus protein) expression.^40^ In fact, the same study showed that of 126 patients with *FGFR1* mRNA overexpression, only 6 concordantly had *FGFR1* amplification. Of these six patients, four received treatment with ponatinib, which was poorly tolerated. Therefore, *FGFR1* overexpression appears to occur independently of gene amplification in lung cancer and potentially other cancer types, supporting the future inclusion of FGFR mRNA and protein levels in fusion-negative cases as potential screening biomarkers into clinical trial design of FGFR-targeted therapy.

In summary, *FGFR1–4* genomic alterations are highly diverse and present at low to moderate frequencies across many tumour types. Thus, comprehensively cataloguing and characterising these diverse alterations has the potential to further benefit cancer patients as the use of FGFR inhibitors in patients with *FGFR* fusion-driven cancers has led to durable responses and improved survival (outlined below).

## Diagnostic approaches to detect *FGFR* genomic alterations

The detection of somatic genomic alterations in *FGFR* is complex, given the variety of *FGFR* alterations observed, including SNVs, gene fusions and CNAs. NGS approaches are the most versatile for detecting these events from tumours or circulating tumour DNA (ctDNA) fragments. Although most methods are suitable and accurate for the detection of SNVs and CNAs, not all methods are equally effective for detecting gene fusions, which are the most challenging from a diagnostics perspective due to the complex nature of rearrangements. Thus, we will focus on the challenges associated with detecting *FGFR* gene fusions.

### DNA-based approaches

Gene fusions involving *FGFR* were first described in haematological malignancies, such as multiple myeloma and 8p11 myeloproliferative disorders through cytogenetics and fluorescence in situ hybridisation-based approaches.^41,42^ Following the advent and application of NGS approaches, *FGFR* fusions were subsequently discovered in solid tumours. As NGS technologies were first translated into novel molecular diagnostics in the clinic, DNA-sequencing of introns containing common gene fusion breakpoints emerged as the first strategy for fusion detection because of the ease of access to suitable quality DNA from formalin-fixed, paraffin-embedded tumour specimens (Table 1). Targeted DNA-sequencing assays utilise hybridisation-based capture for the detection of selected introns known to be involved in gene fusions. Thus, these assays are limited to selected genes and introns and are unable to detect fusions involving novel intronic breakpoints. These approaches have been particularly well studied for rearrangements involving oncogenes, including *ALK*, *RET* and *ROS1*. An important limitation of DNA-based approaches is that some introns are either very large (up to 1,000,000 bp) or contain very repetitive regions that are purposefully not targeted. Consequently, some DNA assays do not fully capture these intronic regions and can miss certain fusions. Although any gene fusion can potentially be missed, fusions involving genes with large introns (*NTRK*) or introns with numerous repetitive elements (ROS1 and FGFR) are especially susceptible to being missed.^43^ Thus, transcriptome or RNA-sequencing (RNAseq) approaches have emerged as important alternatives for the discovery of novel gene fusions or rearrangements.^44^

**Table 1:** Diagnostic Approaches for *FGFR* Fusion Detection.

Source/analyte	Approach	Example	FGFR specific?	Novel fusion partner?	Novel fusion breakpoint	Sequencing bandwidth?	Gene expression?	FFPE tissue/other
Tumour tissue/DNA	FISH	FGFR1 FISH (Mayo, KDL^a^)	✓	✓^b^	✓^b^	N/A	?	✓ FFPE tissue
		FGFR2 FISH (Mayo)						
	Intronic hybridisation/capture	FoundationOne	?	✓^c^	?	High ($$$)	?	✓ FFPE tissue
		MSK-IMPACT						
Tumour tissue/RNA	RT-PCR	QIAGEN *therascreen*®	✓	?	?	N/A	?	✓ FFPE tissue
	Multiplexed amplicons	GeneTrails© Comprehensive Solid Tumour Panel	?	?	?	Low ($)	✓	✓ FFPE tissue
	Anchored multiplex PCR	Archer FusionPlex Solid Tumour	?	✓	?	Low ($)	✓	✓ FFPE tissue
	Exonic hybridisation/capture	OSU-SpARKFuse	?	✓	✓	Moderate ($$)	✓	✓ FFPE tissue
		Caris						
		Tempus						
		FoundationHeme						
Blood/cell-free DNA	Circulating tumour DNA	Guardant360	?	✓^c^	?	High ($$$)	?	✓ Serial monitoring of FGFR alterations
		FoundationACT						*x* Ambiguous sensitivity
		MSK-ACCESS						

Dollar signs ($) associated with sequencing bandwidth indicate relative cost of sequencing ($$$ = most expensive, $$ = moderately expensive, $ = least expensive).

*FFPE* formalin-fixed paraffin-embedded, *FISH* fluorescence in situ hybridisation, *MSK-ACCESS* Memorial Sloan Kettering-analysis of circulating cell-free DNA to evaluate somatic status, *MSK-IMPACT* Memorial Sloan Kettering-integrated mutation profiling of actionable cancer targets, *RT-PCR* reverse-transcription polymerase chain reaction.

^a^Knight Diagnostics Laboratories.

^b^Break-apart FISH probes are capable of detecting gene fusions, but do not identify specific fusion partners or breakpoints. Break-apart FISH also has limited spatial resolution and can miss small inversions close to the probes.

^c^Can miss known and novel gene fusions due to incomplete targeting of introns because of repetitive elements or large size.

### RNA-based approaches

Due to the inherent challenges of detecting gene fusions through DNA-based assays, several groups have turned to RNA-based diagnostic approaches to detect gene fusions and these approaches are becoming more widespread.^45,46^ In a clinical laboratory setting, RNA-based testing presents several challenges, such as quality of tumour tissue, quality/quantity of RNA extracted and complex analysis. Since gene fusions are not native to the human genome, trying to match sequences containing fusion reads back to the genome proves to be challenging and requires the implementation of specialised tools. RNA approaches can be polymerase chain reaction (PCR)-based for known fusions and exons (e.g. anchored multiplex PCR) or can be hybridisation-capture-based to evaluate the whole transcript (e.g. RNAseq with hybridisation capture). In 2019, QIAGEN launched *therascreen®* FGFR, the first FDA-approved companion diagnostic for FGFR alterations in urothelial cancer. This RNA-based test enables the qualitative detection of two-point mutations in exon 7 (*R248C* and *S249C*), two-point mutations in exon 10 (G370C and Y373C), and two fusions (*FGFR3–TACC3v1* and *FGFR3–TACC3v3*) in *FGFR3* using RNA derived from formalin-fixed, paraffin embedded urothelial tumour tissue. Although PCR-based assays are very sensitive for fusion detection, they are limited to exons that encompass previously identified fusion breakpoints and are, therefore, unable to detect novel fusions. Hybridisation-capture-based techniques are the least biased and most likely to be effective for discovering novel fusions. However, these assays are accompanied by an increased complexity of bioinformatics analysis, which involves extensive filtering for false-positive events. The implementation of these assays is further limited by the scarcity of positive controls for analytic validations to determine sensitivity and specificity, which is of particular importance for *FGFR* genes, as there is a great diversity in fusion partners (>300 unique genes).^12^ This diversity appears to be specific for *FGFR*, as other genes commonly involved in fusions, such as *ALK*, have significantly fewer unique partners (~30 unique genes).^47^

The advantages of RNA-based detection approaches for gene fusions were demonstrated in a cohort of patients with lung cancer, who underwent testing with a DNA-based NGS assay (Memorial Sloan Kettering-Integrated Mutation Profiling of Actionable Cancer Targets (MSK-IMPACT)).^43^ Benayed et al.^43^ evaluated a group of 2522 lung adenocarcinomas with MSK-IMPACT and identified 254 cases that were negative for known driver mutations and had sufficient material for RNAseq. Next, they applied an anchored multiplex PCR amplicon assay, which revealed previously undetected fusions in 29 out of 232 evaluable cases (22 cases experienced a technical failure). Importantly, 27 of these fusions were actionable with targeted therapy. Nearly half of the gene fusions missed by MSK-IMPACT were expected to be detected based on the assay design, highlighting the limitations of intronic-DNA-based sequencing for fusion detection. RNA-based approaches are able to overcome many of these limitations and are our preferred method for fusion detection given the potential clinical impact for patients.

### Analysis of ctDNA

In addition to genomic testing using tumour tissues, liquid biopsy-based approaches that evaluate ctDNA have emerged as a means to detect tumour-specific genomic alterations, including gene fusions. These DNA fragments are small, ranging from 90 to 150 bp.^48^ The amount of ctDNA present varies and is thought to depend on a patient’s tumour burden, location and vascularity of metastasis, as well as the previous use of therapies that can lyse or reduce tumours, all of which can affect the representation of ctDNA fragments in a patient’s blood. Thus, findings from ctDNA analysis are generally considered specific, but might not completely represent the diversity present across metastatic disease sites. The accuracy of the use of ctDNA assays for *FGFR* gene fusions is not clear, but there is evidence that existing assays might show a reduced sensitivity for fusions. For instance, when a commercial ctDNA assay (Guardant360) was applied to tumour tissues from 14 patients, which harboured 20 unique *FGFR2/3* alterations, the assay was able to detect 4 out of 5 SNVs, 1 out of 2 amplifications and only 5 out of 13 fusions.^49^ Guardant360 is also being used for the identification of resistance mutations in patients being treated with FGFR inhibitors. The authors of a study evaluating seven patients with *FGFR2*-fusion-positive cholangiocarcinoma using the ultra-deep ctDNA panel Memorial Sloan Kettering-Analysis of Circulating Cell-free DNA to Evaluate Somatic Status (MSK-ACCESS) reported the detection of 19 acquired point mutations across five patients who progressed on targeted FGFR therapy.^50^ In summary, these preliminary studies suggest that gene fusions are harder to accurately detect than SNVs and amplifications using existing ctDNA assays. For patients with cancers that are postulated to have *FGFR* fusions, we recommend tumour tissue testing for clinical care and we suggest that ctDNA should be reserved for research purposes for the time being.

## FGFR-targeted therapies

**Fig. 2:**
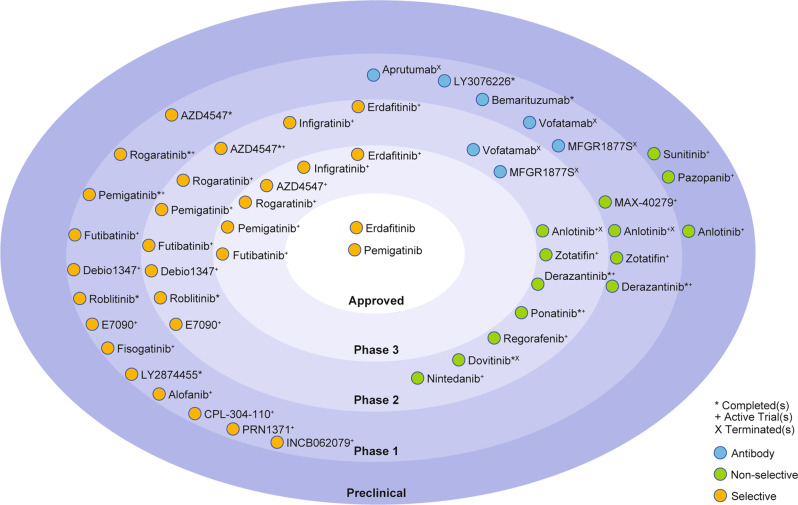
Current clinical landscape of FGFR inhibitors. Numerous FGFR inhibitors are currently being assessed in preclinical, Phase 1, Phase 2 and Phase 3 clinical trials. This figure does not include trials assessing FGFR inhibitors in combination with other therapeutic strategies. Erdafitinib and pemigatinib are currently the only approved inhibitors for use in the treatment of patients with *FGFR*-altered urothelial cancers and cholangiocarcinoma, respectively.

Given the wide spectrum of *FGFR* genomic alterations observed across multiple cancer types, it is perhaps not surprising that numerous FGFR-directed therapies are currently being evaluated in preclinical and clinical studies (Fig. 2). The current landscape of FGFR inhibitors includes small-molecule receptor TKIs (non-selective, selective and covalent), monoclonal antibodies, FGF ligand traps and DNA/RNA aptamers.

### Non-selective TKIs

Initial clinical studies to inhibit FGFR used multitargeted kinase inhibitors, including ponatinib, dovitinib and nintedanib, which, although not designed to target FGFR specifically, are able to reversibly and competitively bind to, and therefore disrupt, the ATP-binding pocket in FGFR1–4. For example, dovitinib (TKI258) shows inhibitory activity against VEGFR1–3, platelet-derived growth factor receptor-β, FGFR1–3, FLT-3, KIT, RET, TrkA and colony-stimulating factor-1.^51^ However, as with most non-selective inhibitors, toxicity remains a significant barrier to the clinical use of dovitinib,^51^ although non-selective FGFR inhibitors might overcome therapy-related secondary mutations that lead to treatment resistance. Ponatinib, for instance, has demonstrated activity against the BCR–ABL T315I gatekeeper mutation that confers resistance to imatinib in patients with chronic myeloid leukaemia and is currently approved for the treatment of this patient population.^52^ Other non-selective TKIs that are currently being assessed in clinical trials for FGFR-mutant cancers include nintedanib, regorafenib, derazantinib, zotatifin, anlotinib, MAX-40279, pazopanib and sunitinib.^53–60^

### Selective TKIs

Most toxicities related to the use of non-selective FGFR inhibitors are attributed to the homology of the ATP-binding domain across the human kinome.^61^ The promise shown from preclinical studies targeting FGFR, however, has led to the development of FGFR-specific (reversible) TKIs. The first generation of these TKIs aimed to target FGFR1–4 (pan-FGFR inhibitors) and included AZD4547, erdafitinib (JNJ-42756493), Debio1347, infigratinib (BGJ398), LY2874455, E7090, pemigatinib, CPL-304-110 and rogaratinib (BAY1163877) (Fig. 2). Most of these pan-FGFR inhibitors, however, fall short in their ability to competitively bind FGFR4 due to its structural dissimilarity to FGFR1–3, with LY287445 and erdafitinib being notable exceptions.^62,63^ Alofanib is yet another selective inhibitor of FGFR2, but utilises an allosteric mechanism to block FGFR2 signalling.^64^

The development of pan-FGFR inhibitors continues to move towards increased selectivity and stronger binding kinetics. The development of irreversible inhibitors has seen increased attention over the past few years. These inhibitors form a covalent bond and cannot be readily displaced by ATP, resulting in prolonged inhibition.^65^ Futibatinib (TAS-120), a novel pan-FGFR therapy currently being evaluated in Phase 1/2 clinical trials, forms a covalent adduct with a cysteine side chain within the phosphate-binding loop of FGFR.^66^ Other covalent inhibitors (including fisogatinib (BLU-554), roblitinib (FGF401), INCB062079 and PRN1371) all use similar irreversible binding strategies, but almost exclusively target FGFR4.^67^

### Monoclonal antibodies and ADCs

Monoclonal antibodies represent another class of selective inhibitors that, in the case of FGFR, function through a number of mechanisms, including disruption of ligand binding and/or receptor dimerisation, or conjugation of the antibody of interest to a cytotoxic agent (ADCs). Early screening studies identified GP369 as a highly specific anti-FGFR2 antibody capable of suppressing FGFR2-driven cell growth both in vitro and in vivo.^68^ BAY 1187982 (aprutumab ixadotin) is an ADC that uses a derivative of the highly potent microtubule-disrupting agent auristatin and is selective for the FGFR2-IIIb and FGFR2-IIIc isoforms (*FGFR1–4* undergo extensive alternative splicing). Preclinical studies showed that treatment with BAY 1187982 resulted in dose-dependent tumour regression in both triple-negative breast cancer and gastric cancer xenograft models with *FGFR2* overexpression.^69^ Although a Phase 1 trial for BAY 1187982 in patients with advanced solid tumours expressing *FGFR2* was opened in 2015, the drug was poorly tolerated and the maximum-tolerated dose was below the estimated therapeutic threshold, resulting in the early termination of this first-in-human study.^70^ The most clinically promising FGFR2 monoclonal antibody currently in development is bemarituzumab (FPA144), which specifically targets FGFR2-IIIb and is glycoengineered to enhance antibody-dependent cell-mediated toxicity (ADCC), a process whereby effector immune cells recognise and kill target cells that display the antibody. Favourable safety and activity were observed in a Phase 1 study and a Phase 3 study (FIGHT) evaluating standard of care chemotherapy in combination with FPA144 or placebo as first-line therapy for patients with gastric cancer overexpressing FGFR2 and is currently open for enrolment (NCT03694522).^71^

Antibody therapies targeting FGFR3 are also currently undergoing clinical development. MFGR1877S binds to FGFR3 with high affinity to competitively inhibit native ligand binding and prevent receptor dimerisation, not only in cells with wild-type *FGFR3* but also in cells with the most prevalent cancer-associated mutants of *FGFR3.*^72^ In addition, MFGR1877S was shown to induce ADCC using in vitro models of multiple myeloma. Phase 1 clinical trials have been completed in multiple myeloma patients with the t(4; 14) translocation causing overexpression of FGFR3 (NCT01122875) and advanced solid tumours (NCT01363024). MFGR1877S was well tolerated by patients on both studies and stable disease (SD) was the best response achieved (6/14 myeloma patients and 9/26 patients on the solid tumour study, including 5 patients with urothelial carcinoma, 2 patients with adenoid cystic carcinoma and 2 patients with carcinoid tumours).^73,74^ LY3076226 is an FGFR3-specific ADC that has DM4 (ravtansine), a maytansine derivative, as the cytotoxic agent.^75^ In a lung cancer patient-derived xenograft (PDX) model driven by an *FGFR3–TACC3* fusion, treatment with LY3076226 resulted in a durable complete response in 100% of mice.^76^ In addition, tumour stasis or regression was observed in bladder cancer PDX models harbouring *G370C*, *S249C* or *R248C FGFR3* mutations treated with LY3076226.^76^ A Phase 1 study with LY3076226 in patients with advanced or metastatic cancer was completed in 2018 and results are still pending (NCT02529553). Vofatamab (B-701) is yet another promising FGFR3 monoclonal antibody in clinical development, and was shown to be well tolerated in combination with docetaxel in patients with urothelial cell carcinoma in the FIERCE-21 study (NCT02041542).^77^ Not surprisingly, enhanced activity was observed in patients with *FGFR3* mutations or fusions compared with wild-type patients. However, preliminary data from the FIERCE-22 study, which combines vofatamab with the immune checkpoint inhibitor pembrolizumab in metastatic urothelial carcinoma show benefit even in FGFR3 wild-type patients compared with previous studies of pembrolizumab alone (NCT03123055).^78^

### FGF ligand traps

Another approach to inhibiting FGF/FGFR signalling in cancer involves disrupting the binding of FGF ligands to their cognate receptors using FGF ‘traps’. One strategy for these FGF traps is the development of decoy receptors that lack the transmembrane and cytoplasmic domains, but maintain the extracellular FGFR domain, which allows for interaction with, and consequent sequestration of, FGF ligands. FP-1039/GSK3052230 is a soluble decoy receptor comprising the extracellular region of FGFR1 fused to the human IgG1 Fc fragment. FP-1039 demonstrated efficacy against *FGFR2* mutant endometrial cancer cells and *FGFR1* amplified lung cancer cells in vivo in preclinical studies.^79^ A Phase 1 study of FP-1039 in non-selected cancer patients was well tolerated and demonstrated toxicities typically associated with FGFR TKIs, including hyperphosphatemia and retinal changes, although nail and skin toxicities were not observed.^80^ Further studies combining FP-1039 with pemetrexed and cisplatin in mesothelioma patients and with paclitaxel and carboplatin in patients with *FGFR1*-amplified non-small cell lung cancer have also been completed.^81,82^

## Clinical outcomes for patients receiving FGFR inhibitors

Commensurate with the surge in the development of diverse therapies (inhibitors, ligand traps, antibodies) directed against FGFR signalling in cancer, there are currently numerous clinical trials, ranging from early phase to large randomised Phase 3 studies, involving FGFR inhibitors (as monotherapy or in combination) for a wide spectrum of solid cancer types. In addition to the direct anti-tumour effects of suppressing cell growth and proliferation exerted by FGFR inhibition, it is possible that the clinical response seen with FGFR inhibitors may also be an indirect effect of the suppression of aberrant angiogenesis. A search on ClinicalTrials.gov on January 16, 2020, for ‘Interventional’ clinical trials with the filters ‘recruiting; not yet recruiting; or active, not recruiting’ uncovered 121 studies. Early phase trials that combine a specific FGFR inhibitor with chemotherapy or immunotherapy are also ongoing and include pemigatinib combined with gemcitabine/cisplatin, pembrolizumab, docetaxel or trastuzumab (NCT02393248), and rogaratinib combined with atezolizumab (NCT03473756).

The most robust data on anti-tumour activity and efficacy have been generated from trials using pan-FGFR inhibitors—specifically, trials in urothelial cancers with *FGFR3* mutations and iCCA with *FGFR2* fusions, as described below.

### FGFR inhibitors in the treatment of urothelial cancer

In a pivotal Phase 2 study of erdafitinib (BALVERSA, Janssen Pharmaceutical Companies) in 99 patients with *FGFR*-altered urothelial cancers, 74 of whom had *FGFR3* mutations and 25 of whom had *FGFR2/3* fusions, an objective response rate (ORR) of 40% was demonstrated with a median duration of response (DOR) of 5.6 months. Furthermore, 39% of patients demonstrated SD. Median progression-free survival (PFS) was 5.5 months and median overall survival (OS) was 13.8 months. These results led to the accelerated approval of erdafitinib by the FDA in April 2019 for previously treated *FGFR3*-altered urothelial carcinomas.^10^ Additional pan-FGFR inhibitors that have been studied in urothelial cancer include infigratinib (QED Therapeutics), pemigatinib (Incyte Corporation) and rogaratinib (Bayer). In a Phase 1 expansion cohort study of infigratinib that enrolled 67 patients with *FGFR3* alterations, an ORR of 25% and an SD of 39% were reached.^83^ Compared with erdafitinib, infigratinib had slightly inferior median PFS and OS values of 3.7 and 7.7 months, respectively, potentially due to enrolling slightly later-line patients. A Phase 2 study of pemigatinib conducted in 103 *FGFR*-altered patients (comprising 61 patients with *FGFR3* mutations or fusions and 42 patients with other *FGFR/FGF* driver alterations) achieved an ORR of 21% and a SD of 36%, with a median PFS of 4.1 months (OS not assessed).^84^ Finally, a Phase 1 expansion study of rogaritinib was carried out in 52 urothelial cancer patients with high *FGFR1–3* mRNA expression in pre-treatment tumour biopsies.^85^ Of these 52 patients, 48 harboured high *FGFR3* expression in their tumours (16/48 had *FGFR3* mutations; 2/48 had *FGFR3* fusions); an ORR of 24% and SD of 49% were achieved, with median PFS of ~3.3 months.

### FGFR inhibitors in the treatment of cholangiocarcinoma

The results from numerous clinical trials in patients with *FGFR*-altered cholangiocarcinoma demonstrate a similar activity and efficacy for FGFR inhibitors in this rare cancer type to those in urothelial cancer. Unlike urothelial cancer, which has a high rate of *FGFR3* mutations, cholangiocarcinoma harbours an increased frequency of fusions or rearrangements that favour *FGFR2* and a diverse group of partner genes. Although pemigatinib is currently the only FDA-approved  FGFR inhibitor for the treatment of advanced iCCA with *FGFR2* genomic alterations, additional approvals will likely be forthcoming, given data from multiple therapeutic trials. For example, in a Phase 2 study of infigratinib in 71 cholangiocarcinoma patients with *FGFR2* fusions, an ORR of 27% and SD of 58% were observed, with a median PFS and OS of 6.8 and 12.5 months, respectively.^86^ Leading to its approval,  a Phase 2 study of pemigatinib in cholangiocarcinoma patients showed an ORR of 35.5% and median DOR of 7.5 months and led to median PFS and OS of 9.2 and 15.8 months, respectively, in one of three cohorts, which comprised 107 patients with only *FGFR2* fusions.^87^ A Phase 2 study of derazantinib (Basilea) in 29 patients with *FGFR2*-fusion-positive cholangiocarcinoma demonstrated an ORR of 21% and SD of 62%, with a median PFS of 5.7 months (median OS not yet reached after a median follow-up time of 20 months).^55^ Erdafitinib has demonstrated efficacy in therapeutic trials for cholangiocarcinoma patients with *FGFR2* fusions, similar to results in patients with urothelial carcinoma with *FGFR3* mutations, although the cholangiocarcinoma trials contained fewer patients.^88,89^ Finally, the drug futibatinib (Taiho Oncology)—a covalent, irreversible inhibitor of FGFR1–4—is the only FGFR inhibitor to have shown limited efficacy in cholangiocarcinoma patients previously treated with a different (reversible) FGFR-TKI.^90^ Phase 1 data showed that, of the 13 patients who had progressive disease on one or more FGFR inhibitors, treatment with futibatinib led to an ORR in 4 of these patients (31%)—three out of these four patients had an *FGFR2* fusion, one had an *FGFR2* amplification.^91^ Of the total of 28 patients with *FGFR2* fusions, 20 demonstrated tumour shrinkage and 7 (25%) achieved confirmed partial responses.^91^ Of note, two Phase 3 clinical trials are now underway to compare the efficacy of infigratinib (NCT03773302) and pemigatinib (NCT03656536) versus standard-of-care chemotherapy gemcitabine/cisplatin for the first-line treatment of advanced or metastatic cholangiocarcinoma with *FGFR2* gene rearrangements.

### Tumour-agnostic treatment

It has become clear that abrogating aberrant FGFR signalling in urothelial cancer (driven by *FGFR3* mutations) and cholangiocarcinoma (driven by *FGFR2* fusions) has led to improved clinical outcomes in these molecularly defined patient populations. Efficacy data from these trials provided the rationale for the development of multiple genomics-driven trials^92^ that aim to determine the anti-tumour activity of various FGFR inhibitors (e.g. pemigatinib, Debio1347, futibatinib and infigratinib) independent of cancer histology. Results from these genomics-driven trials will help to further delineate whether *FGFR* gene family amplification (the most common type of *FGFR* alteration in human cancers), *FGFR* overexpression or the presence of specific subsets of activating mutations could represent alternative viable biomarkers to gene fusions/rearrangements for FGFR inhibitor therapy. In addition to achieving a precise molecular stratification of patients who will benefit from FGFR inhibitor therapy, the infigratinib basket study also intends to evaluate the efficacy of infigratinib in patients who have progressed on prior FGFR inhibitors (NCT04233567). Although erdafitinib and pemigatinib currently  are the only FDA-approved FGFR inhibitors with a histology-specific indications (i.e. urothelial cancer and cholangiocarcinoma), it is likely that one or more drugs in this class will eventually achieve tumour-agnostic labels, as well as additional histology-directed indications, in the upcoming decade. Finally, it is worthy to note that the response rates seen with FGFR inhibitor therapy in multiple cancer histologies (~20–40%) fall short of response rates seen for patients with other oncogenic fusions, such as those involving *ALK*, *ROS1*, and *NTRK* (~60–70%). Many patients when treated with FGFR inhibitors demonstrate stability of their cancer. This may be due several factors, including degree of oncogene addiction, presence of a downstream promiscuous signalling network employed by the fusion FGFR receptor (indeed, FGFR2 has >300 unique fusion partners) or compensatory upregulation of pro-tumorigenic pathways upon FGFR inhibition.^12,39^ Although studies have investigated acquired mutations after prolonged treatment with FGFR inhibitors, the immediate signalling changes that occur shortly after TKI initiation are not currently known. Therefore, enhancing the response to FGFR inhibitors may need additional robust pre-clinical studies and correlatives to evaluate signalling pathways that can be co-targeted upfront.

### FGFR inhibitors and toxicity

The unique spectrum of toxicities associated with pan-FGFR inhibitors results from the blockade of physiological functions of FGF/FGFR signalling.^93–95^ Significantly, as a class, these drugs lead to disrupted phosphate homeostasis, a process normally mediated by FGF23,^93^ leading to hyperphosphataemia shortly after treatment initiation in a majority of patients. Additional common side effects include stomatitis/mucositis, fatigue, hand-foot syndrome, gastrointestinal events, and ocular events, such as central serous retinopathy. Interestingly, nail events such as onychodystrophy and onycholysis were reported for both erdafitinib and infigratinib, but not for other FGFR inhibitors, including pemigatinib and rogaratinib. Dose-limiting toxicities globally were stomatitis, nail events and fatigue, necessitating dose interruptions or reductions and implementation of supportive therapies. The anticipation of near-future regulatory approval for multiple clinical indications should spur efforts to continuously improve and refine the clinical management of toxicities associated with FGFR inhibitors. Furthermore, the development of agents, such as vofatmab,^84^ a monoclonal antibody that specifically targets FGFR3, and fisogatinib,^96^ an FGFR4 inhibitor, might lead to improved toxicity profiles compared with pan-FGFR inhibitors.

## Mechanisms of resistance to FGFR inhibitors

Resistance to drug therapies can be classified as either primary or secondary. Primary resistance describes an initial lack of treatment response, while secondary (or acquired) resistance describes disease progression after an initial response to treatment and has emerged as a limiting factor to the long-term efficacy of FGFR-targeted therapies. Secondary resistance to FGFR TKIs develops as a result of secondary ‘gatekeeper’ mutations within the FGFR protein or through hyperactivation of alternate mitogenic signalling pathways.

**Fig. 3:**
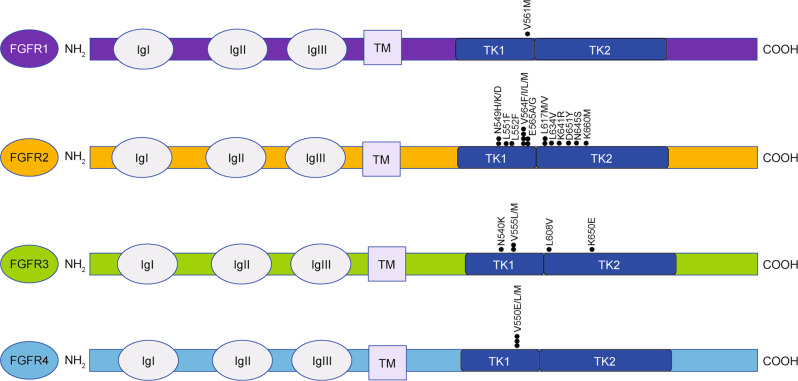
FGFR resistance mutations. Summary of mutations in FGFR1–4 that have been shown to confer resistance to FGFR inhibitors. Ig immunoglobulin domain, TK tyrosine kinase.

In vitro and in vivo studies have shown that mutations occurring at gatekeeper residues in FGFR, such as *FGFR1 V561M* and *FGFR2 V565I*, lead to steric hindrance within the ATP-binding pocket, which precludes the entry and binding of multiple FGFR inhibitors^67^ (Fig. 3). In in vitro studies, infigratinib treatment of Ba/F3 cells containing a TEL–FGFR3 fusion drove the development a number of resistance mutations in the FGFR3 component: *V555L*, *V555M*, *N540K*, *L608V* and *K650E.*^97^ Clinical studies detected the SNVs *FGFR2 V564F*, *N549H*, *N549K*, *E565A*, *K659M*, *K641R* and *L617V* in FGFR2-fusion-positive iCCA patients following treatment with infigratinib and the emergence of these mutations correlated with cancer progression.^97,98^ The *FGFR2 N549H* secondary mutation was also detected in an iCCA patient who developed resistance to pemigatinib.^99^ Moreover, the mutations V550L and V550E in the FGFR4 kinase domain have been demonstrated to confer clinical resistance to erdafitinib in rhabdomyosarcoma, while V550M is associated with erdafitinib resistance in breast cancer.^100^ Another resistance SNV is *FGFR2 V564M*, which appears to confer resistance to infigratinib and dovitinib.^67^

We also observed nine novel *FGFR*2 SNVs (in addition to others that have been previously reported) in a patient with an *FGFR2–BICC1* fusion treated with infigratinib: *D651Y*, *E566G*, *L551/2F*, *L551F*, *L617M*, *L634V*, *N550D*, *N550K*, *N653S* and *V564L* (unpublished data). In another case, a patient presenting with an *FGFR2–SORBS1* gene fusion achieved a maximum tumour burden reduction of 68% from baseline to third-line treatment with the pan-FGFR inhibitor futibatinib after developing resistance to infigratinib and Debio 1347. ctDNA collected at the time of disease progression on second-line treatment was sequenced, and *FGFR2* kinase domain mutations *K660M*, *L618V*, *N550K*, *V565F* and *K715R* were detected.^90^ Although the *K715R* mutation was sensitive to infigratinib in vitro, the *K660M* mutation exhibited a 30-fold increase in IC_50_ (half-maximal inhibitory concentration) value.^90^

In contrast to these well-defined resistance mechanisms, the underlying cause of resistance to current, clinically relevant multitarget kinase inhibitors is far less understood. Within the *FGFR2* gene, mutations such as *M536I*, *M538I*, *I548V* and *L618M* have been shown through in vitro experiments to confer resistance to drugs like dovitinib.^101^ However, due to the overall lack of significant clinical activity and high levels of toxicity, dovitinib is no longer being used clinically.^102^ Additional clinical trials are continuing to investigate whether other non-selective inhibitors, like ponatinib, can be used in patients who have developed resistance to selective FGFR inhibitors.

The development of acquired resistance is not limited to the acquisition of specific mutations, but can also occur through ‘bypass’ signalling—in this case, a non-FGFR RTK signalling pathway might become activated to bypass TKI-mediated inhibition of the FGFR signalling axis. For example, upregulation of the MET signalling pathway leading to re-activation of the ERK/MAPK pathway was observed in conjunction with the development of resistance to infigratinib in *FGFR1*-amplified DMS114 lung cancer cells.^103^ A functional CRISPR/Cas9 screen has also identified additional non-FGFR RTKs, including integrin-linked kinase, SRC, ERBB2 (another member of the EGFR family) and epidermal growth factor receptor, that influence FGFR sensitivity to inhibitors.^104^ As molecular studies begin to more precisely define the mechanisms of acquired resistance, targeting these pathways upfront, in combination with an FGFR inhibitor, might theoretically increase time-to-progression, albeit at the risk of increased toxicities.^98,99^ For instance, based on positive results from preclinical studies targeting both FGFR and phosphatidylinositol-3-kinase (PI3K)/mammalian target of rapamycin (mTOR), the combination of rogaratinib with copanlisib (a PI3K inhibitor) is being evaluated in a dose-expansion cohort of urothelial cancer patients with *FGFR1–4* overexpression who have developed resistance to AZD4547, erdafitinib, pontatinib, dotivtinib or E810.^98,105^ In another study, the deletion of *PTEN* has been shown to confer resistance to FGFR inhibition by activating downstream PI3K/AKT/mTOR signalling.^105^ In addition, previous findings have shown that FGFR inhibition with AZD4547 and EGFR inhibition with cetuximab synergistically inhibited the growth of *FGFR2*-amplified gastric cancer cells.^106^ Conversely, resistance to ERBB2-directed therapy (e.g. trastuzumab) has been shown to be driven by increased FGFR signalling.^107^ This evidence supporting the synergistic signalling between EGFR and FGFR kinase family members again demonstrates the potential use of combination therapy strategies in patients to elicit deeper and more durable responses.

## Conclusions and perspectives

Targeting the FGFR signalling pathway constitutes another successful example of precision oncology enabled by biomarker-driven patient selection and therapies. A variety of *FGFR* alterations have been shown to contribute to oncogenesis. To date, clinical trials have demonstrated that patients with non-urothelial cancers harbouring gene fusions of *FGFR2* or *FGFR3* achieve favourable clinical outcomes when treated with various FGFR inhibitors and that, as urothelial cancer patients have an increased frequency of *FGFR3* point mutations, they tend to respond better to TKI therapy. The data showing clinical benefits are less robust for cancers that have *FGFR* amplifications, SNVs outside important functional domains (e.g. kinase) of FGFR and for haematological malignancies. Importantly, research is actively ongoing to identify and validate the catalogue of ‘driver’ SNVs in *FGFR1–4* in fusion-negative cancers. The goal would be to determine whether these drivers, much like fusion events, might also confer a therapeutic vulnerability to anti-FGFR therapies. A second research emphasis relates to the development of clinical-grade NGS diagnostic tests for the detection of *FGFR* fusions and SNVs using tissue and ctDNA. An assay optimised for the use of ctDNA would improve time-to-detection of driver alterations (given adequate tumour burden), thus enabling the rapid identification of patients for targeted therapies and the real-time detection of acquired mutations that signal impending treatment resistance and cancer progression. Finally, a third area of research emphasis with almost immediate translational impact comprises the study of acquired resistance to FGFR inhibitors. Acquired resistance can occur via tumour-intrinsic mechanisms, including the development of secondary kinase or ‘gatekeeper’ mutations in *FGFR* and/or by the emergence of ‘bypass’ signalling. The role of tumour-extrinsic mechanisms, including whether, when and how the tumour and/or immune microenvironment might be contributing to acquired resistance, is currently underexplored, but is an important knowledge gap that needs to be filled. Given that certain studies have indicated a role for altered FGFR signalling in immune evasion, research into tumour–stroma interactions might reveal insights into whether combination strategies that incorporate immune checkpoint inhibition would represent a viable strategy to overcome acquired resistance or perhaps even be used as a front-line therapeutic strategy in these patients. Ultimately, we believe that research addressing these areas of interest will lead to a deeper understanding of FGFR biology that can subsequently be exploited to improve patient care and outcomes.

## Data Availability

All data are included in the review article.
